# Thriving or Striving: Comparing Intra-Uterine Growth Restricted, Low Birth Weight and Normal Birth Weight Piglets within the First 24 Hours

**DOI:** 10.3390/ani14172508

**Published:** 2024-08-29

**Authors:** Marlotte Loyens, Lieselotte Van Bockstal, Sara Prims, Steven Van Cruchten, Chris Van Ginneken

**Affiliations:** Comparative Perinatal Development, Department of Veterinary Sciences, Faculty of Biomedical, Pharmaceutical and Veterinary Sciences, University of Antwerp, 2610 Wilrijk, Belgium; marlotte.loyens@uantwerpen.be (M.L.); lieselotte.vanbockstal@uantwerpen.be (L.V.B.); sara.prims@uantwerpen.be (S.P.); steven.vancruchten@uantwerpen.be (S.V.C.)

**Keywords:** intra-uterine growth restriction, survival, birth weight, pig, rectal temperature, colostrum intake, latency to suckle, vitality score, neonate

## Abstract

**Simple Summary:**

Our study examined the challenges newborn piglets face, comparing those born with growth restriction, those born with low body weight, and those of normal body weight. We aim to understand if their start in life is related to their physical characteristics and whether this affects their chances of survival. We categorized newborn piglets based on their size and physical characteristics immediately after birth. Over three hours, we assigned vitality scores, measured body temperature, and described behaviors such as seeking sow’s milk. We found that piglets with growth restrictions had more difficulty keeping warm and feeding than their counterparts with a low and normal body weight at birth. Consequently, they faced a higher mortality rate within the first 24 h after birth. This suggests they need special care to improve their survival and health. Our research highlights the importance of recognizing and addressing the needs of specifically intra-uterine growth-restricted piglets early on to improve the welfare of these growth-restricted piglets.

**Abstract:**

This observational study explored the early-life challenges of intra-uterine growth restricted (IUGR), low birth body weight (LBW), and normal birth body weight (NBW) piglets. The aim was to understand the impact of birth weight and intra-uterine growth restriction phenotype on neonatal survival and behavior. Based on weight and phenotype, piglets were classified as IUGR (*n* = 32), LBW (*n* = 34), and NBW (*n* = 29) immediately after birth. The piglets were litter- and sex-matched. Vitality scores were assigned based on motor activity and breathing and complemented with an assessment of umbilical cord condition, rectal temperature, crown–rump length (CRL), time to reach the udder, time to suckle, colostrum intake, and weight gain over 24 h. Beyond the lower birth weight, reduced CRL, and higher mortality rate, IUGR piglets faced several other challenges compared with LBW and NBW piglets. Growth-impaired piglets often struggled to engage in early feeding behaviors and displayed consistently lower rectal temperatures at 1, 3 and 24 h after birth. IUGR piglets showed inadequate colostrum intake and weight loss, which were also observed for LBW counterparts. In contrast, no significant differences were observed in vitality scores and umbilical cord conditions across the groups. In conclusion, our findings underscore the impact of intra-uterine growth restriction on neonatal piglets, emphasizing the need for specialized care strategies to improve survival and health outcomes in IUGR.

## 1. Introduction

Farmers strive to enhance the reproductive potential of sows, given that the number of piglets produced per sow per year is an important economic trait [1,2]. This has resulted in the emergence of hyper-prolific sows, giving birth to more piglets than their average number of functional teats [3,4]. Beyond having supernumerary piglets, litters of hyper-prolific sows encounter additional difficulties. Larger litters tend to have a higher prevalence of piglets that experience levels of growth restriction in utero, more piglets born with low birth weight, more variability of birth weights within a litter, and increased pre-weaning mortality rates [3,5,6,7,8,9,10,11]. A study of a UK herd demonstrated the relationship between litter size and early piglet mortality. Specifically, in litters of 14 piglets, there was an average of 1.2 stillbirths and 0.6 deaths before 12 h, whereas in litters of at least 20 piglets, these numbers increased to an average of 4.1 stillbirths and 2.3 deaths before 12 h [12]. These findings indicate that mortality rates rise with litter size, suggesting significant potential for enhancing productivity in pig farming through optimized management strategies for larger litters. Several studies have already been conducted to evaluate the health and physical condition of newborn piglets, aiming to understand the impact of these factors on their chances of survival [1,3,9,11,13,14,15,16,17,18]. The first few weeks of a piglet’s life are critical, with mortality rates reaching 14% by the end of the suckling period [19,20]. Notably, this mortality rate is at its highest during the first three days after birth [18,21]. Therefore, identifying piglets at greater risk of dying during the first days is crucial for determining which piglets would benefit from strategic interventions, ultimately decreasing pre-weaning mortality rates [10,15]. Many studies use birth weight as the most important criterion, as piglets from most modern domestic pig breeds that weigh less than 1 kg at birth are less viable [9,11,13,21,22,23]. In contrast, Douglas et al. [13] discovered that, despite their initial size disadvantage, some low-birth-weight piglets survived and could even compensate for their initial low birth weight. Some even reached the final body weight of their heavier littermates at slaughter without any intervention [13]. This thus warrants a more critical view on birth weight as the sole criterion. Low birth weight in piglets is a multifaceted issue, encompassing not only those naturally small at birth but also those who have experienced intra-uterine growth restriction (IUGR) [5]. IUGR refers to a condition where offspring encounters compromised growth, preventing them from reaching the genetic potential of the fetus and its organs during gestation [24,25]. This compromised growth is a disadvantage for IUGR piglets compared with those born naturally small, and, as a result, IUGR piglets commonly face increased pre-weaning mortality risks [25,26]. Thus, next to birth weight, additional criteria such as piglets’ vigor, behavior, and specific phenotypical characteristics of IUGR (such as a steep, dolphin-like forehead, bulging eyes, and wrinkles perpendicular to the mouth [27]) supported by morphological measurements like ponderal index (PI) and body mass index (BMI) should be considered to estimate survivability in newborns [14,15,18,28]. Therefore, this study expands upon existing literature by assessing vitality at birth and comprehensively analyzing several critical behaviors such as suckling behavior, colostrum intake [5,29], and the maintenance of body temperature [30,31,32] in the first 24 h of life. Distinctive to our study is the integration of these different factors and the direct comparison of IUGR piglets, low birth weight (LBW), and normal birth weight (NBW) piglets. It was hypothesized that the piglets showing the IUGR morphotype show lesser vitality and performance than their low and normal birth weight counterparts. Consequently, these data offer insight into the strengths and weaknesses of each group, highlighting the nuanced spectrum of neonatal vitality and emphasizing the urgent requirement for customized care to narrow the divide between surviving and thriving.

## 2. Materials and Methods

### 2.1. Ethical Approval

The observational study in a conventional pig farm complied with national legislation and European guidelines (2010/63/EC), as confirmed by the Ethical Committee for Animal Experimentation of the University of Antwerp (ECD Non-Animal Experiment ref 2022-71).

### 2.2. Animals

The study was conducted on a commercial pig farm (Van Dijck H&M, Loenhout, Belgium). Sows (Danbred Yorkshire/Landrace breeds or their hybrids) were artificially inseminated using Duroc boar sperm. Two weeks before farrowing, the sows were randomly distributed between two different farrowing stables and housed in individual farrowing crates (2.25 m × 0.65 m) situated within pens (2.90 m × 1.80 m). A corridor separated the farrowing stables, ensuring that no wall was directly exposed to the outside. The pens had a creep area with a heated floor covered by drying powder (Opticomfort Dry Protection, Indufarm, Ingelmunster, Belgium) around the farrowing time. The sows ate a commercial gestation diet (Premium zeugenvoer drachtperiode solide, De Heus, Lommel, Belgium) up to farrowing. After farrowing, the diet was switched to a commercial lactation diet (Premium lacto zeugenvoer comfort, De Heus, Lommel, Belgium). During farrowing, the temperature in the stable was 25–26 °C. Births were initiated by giving an intramuscular injection of cloprostenol (Planate^®^, MSD Animal Health, Boxmeer, The Nederland, 0.0875 mg/mL) on day 115 of gestation. If farrowing had not begun by the start of day 116, the sows received an intramuscular injection of oxytocin (Oxytocin Kela 10IE^®^, Kela, Hoogstraten, Belgium, 10 I.U./mL). Parturitions were monitored on day 116, but interference was kept to a minimum. When the farrowing process took too long, an additional oxytocin injection was given. Following the completion of parturitions, long-acting oxytocin (LongActon^®^, Vetoquinol, Niel, Belgium, 0.07 mg/mL) was administered intramuscularly. All liveborn piglets were subjected to the farm’s standard handling procedures, which include ear-tagging, tail docking, and teeth-clipping within 48 h after birth. These procedures were carried out by trained farm staff to reduce the risk of tail biting and other biting lesions among the piglets in the litter, as well as to minimize injuries to the sow’s teats and the ears or tails of other pigs. It is important to note that only the pointed tips of the teeth were removed, avoiding full removal or cutting down to the gum-line. Tooth reduction was carried out between 20–28 h after birth, and most of the selected piglets had not yet undergone this procedure before the 24 h measurement; only a few had undergone tooth reduction by that time. Tail docking was performed 24–48 h after birth to prevent tail biting lesions in the piglets as they grew. However, none of the selected piglets experienced tail docking during our observation window. While Council Directive 2008/120/EC has outlawed these practices, and the European Food Safety Authority (EFSA) has recommended against them, the farm staff opted for these measures based on veterinary advice to address specific issues related to tail biting, invoking the allowed exception.

### 2.3. Piglet Selection

Immediately after birth, all piglets were weighed to calculate the mean birth weight of the litter. The piglets were allocated into three categories based on birth weight and physical characteristics. The first category, IUGR piglets, exhibits a typical IUGR phenotype characterized by a steep, dolphin-like forehead, bulging eyes, wrinkles perpendicular to the mouth [27] and a birth weight lower than the mean birth weight − 1 standard deviation (SD), of the litter. The second category, low birth weight (LBW) piglets, had a birth weight lower than the mean birth weight − 1 SD, but did not exhibit any characteristics of the IUGR phenotype [33,34]. The third category, normal birth weight (NBW) piglets, was defined as having a birth weight within the mean birth weight ± 1 SD, without IUGR characteristics [21]. Per litter, each IUGR piglet was sex-matched with an NBW piglet and, if possible, an LBW piglet. The selected piglets were ear-tagged for further observation. This study observed a total of 32 NBW (16 males and 16 females), 34 LBW (18 males and 16 females), and 29 IUGR (16 males and 13 females) piglets.

### 2.4. Observational Study

General information, such as that pertaining to sex, birth hour, birth order of the piglets, and litter size, was documented. Every newborn piglet was assigned a vitality score between 0 and 4 based on breathing and activity within 15 s after birth, as described by Baxter et al. (Table 1) [15,17]. The appearance of the umbilical cord was recorded at birth by noting whether it was ruptured or intact. If the umbilical cord ruptures during delivery, this could lead to in utero hypoxia and lower the vitality and survivability of the piglet [35]. Rectal temperature was recorded from the selected piglets at 0 h (within 1 min after birth), 1 h, 3 h, and 24 h after birth using a digital rectal thermometer (Predictor Thermometer Digital Flex; accuracy: ± 0.2 °C; lower limit: 32.0 °C). Additionally, the selected piglets’ body weight at birth (BWB) was documented immediately after recording the rectal temperature at birth using a digital scale (Kern weegplateau, MS Schippers, Hapert, The Nederland, measuring range: 0–60 kg, precision: 20 g). Crown–rump length (CRL) was measured starting from the head’s crown to the tail’s base of the piglets. This way, body mass index (BMI; body weight/CRL^2^), which reflects the relationship between weight and surface area, and ponderal index (PI; body weight/CRL^3^), which assesses the relationship between weight and volume, could be calculated. Furthermore, selected piglets were observed from birth until suckling. Hereby, latency to reach the udder (the time it took from birth until touching the udder with the snout), latency to reach a functional teat (the time it took from birth until touching a teat that is capable of providing milk with the snout) and latency to suckle (the time it took from birth until the moment a piglet starts to show muzzle motions as a sign of suckling) were noted, with a 3 h cutoff for each of these parameters. Finally, the selected piglets were weighed after 24 h to calculate the weight gain and colostrum intake (Cl), as described by Theil et al. [36]:

Cl = −106 + (2.26 × WG) + (200 × BWB) + (0.111 × D) − (1414 × (WG/D)) + (0.0182 × (WG/BWB))

with weight gain (WG, g; body weight at 24 h—body weight at birth), BWB (body weight at birth, kg), and duration of suckling from birth until 24 h after birth (D, min) as variables. The surviving piglets were counted after 24 h to calculate the mortality percentage.

### 2.5. Statistical Analysis

Linear mixed models were fitted in JMP Pro 16 (SAS Institute Inc., Cary, NC, USA) to analyze the observational data. Birth weight category and sex were considered as a fixed effect. In addition, to account for potential interactions, the interaction term between birth weight category and sex was added to the model. When the interaction term was insignificant (*p* > 0.05), it was removed from the starting model. In the absence of interaction, sex differences were examined through the broad variations associated with them instead of evaluating sex differences within the specific weight groups (NBW, LBW and IUGR). Sow was added as a random effect to account for the interdependence among siblings from the same litter. The farrowing round, which refers to the data collection round where each visit corresponds to a different farrowing cycle (first visit as the first farrowing round, second visit as the second round, and so on), was not included in the final model as a random effect since further analysis proved this factor to be insignificant. To comply with the normal distribution and/or homoscedasticity assumption, birth weight and colostrum intake values required a square root transformation; BMI, PI, and weight gain were transformed via a logarithmic transformation; and CRL underwent a square transformation. Due to the data transformation, all data were presented as median (interquartile range; IQR). A differentiation was made between IUGR piglets that were still alive after 24 h (IUGR alive) and those that had died within the same period (IUGR dead). Temperature data were considered repeated measures with as fixed effects birth weight category, time, sex, and all relevant interactions between birth weight category, time and sex. Because repeated measurements were performed on the same piglet, the piglet (nested in the sow) was added as a random effect. Stepwise backward modelling was used to simplify the model so that all non-significant effects were removed from the initial model. Limitations from the rectal thermometer include restricted recording of piglet temperatures below 32 °C. To consider this limitation, any data falling below this detection threshold were substituted with the mean between the detection limit and the lowest temperature observed in a pilot study of five live piglets with temperatures under the 32 °C threshold. This is an adapted method of substituting the data by detection limit [37,38]. The lowest temperature recorded by the thermometer was 26 °C (Amarell^®^ Electron; accuracy: ± 0.1 °C; lower limit: −50.0 °C). Therefore, all recorded temperatures below 32 °C were replaced with the value of 29 °C. As there was a 3 h observation period post-partum, latency times for piglets to reach the udder, find a functional teat, and start suckling were analyzed using Cox’s proportional hazard model [39]. This analysis was extended with a post-hoc analysis of the risk ratios to identify which group reached the udder, found a functional teat, or began suckling significantly quicker. The proportions of the piglets that died within 24 h in each group (NBW, LBW, and IUGR) were calculated to estimate the probability of higher mortality between the different groups. A Fisher’s exact test followed this to compare these proportions between the groups.

Finally, an exploratory principal component analysis (PCA) was conducted on all the variables, including 24 h weight, body weight at birth, weight gain, colostrum intake, BMI, PI, CRL, rectal temperature at birth, 1 h, 3 h, and 24 h post-partum, latency to reach the udder, a functional teat, start suckling, umbilical cord appearance, vitality score, sex, and category. To convert categorical variables into continuous variables, umbilical cord appearance was coded as 0 for an intact umbilical cord and 1 for a ruptured umbilical cord. The category of the piglets (NBW, LBW, or IUGR) was coded into a new variable called category code, where NBW = 1, LBW = 2, and IUGR = 3.

The results were considered statistically significant when the *p*-value was less than 0.05. Post-hoc analysis applying Tukey’s correction was used to compare the different categories when necessary.

## 3. Results

### 3.1. Piglet Characteristics

Altogether, the selected piglets were derived from 28 sows, with an average litter size of 21 ± 4 piglets. Inherent to the selection process, both LBW and IUGR piglets were lighter (*p* < 0.0001) but also shorter (*p* < 0.0001) in comparison with NBW piglets (Table 2). Moreover, there were noticeable and significant differences in both BWB (*p* < 0.0001) and CRL (*p* = 0.010) between LBW and IUGR piglets. This resulted in a lower BMI for LBW and IUGR piglets when compared with NBW piglets (*p* < 0.0001), and significant BMI differences were also evident between the LBW and IUGR piglet groups (*p* = 0.001). The PI was comparable across all groups (*p* = 0.805). Finally, while sex did not significantly impact the BWB (*p* = 0.321) and BMI (*p* = 0.494), it did influence CRL (interaction term: *p* = 0.028) and PI (interaction term: *p* = 0.016). Sex differences were observed solely in the LBW group (CRL: *p* = 0.025; PI: *p* = 0.039), where males had a shorter CRL and, consequently, a higher PI than females.

Over 24 h, 31% of the IUGR piglets died, whereas there were no reported deaths among the NBW or LBW piglets (*p* < 0.0001) (Table 2).

### 3.2. Vitality Score and Umbilical Cord Condition

**Figure 1:**
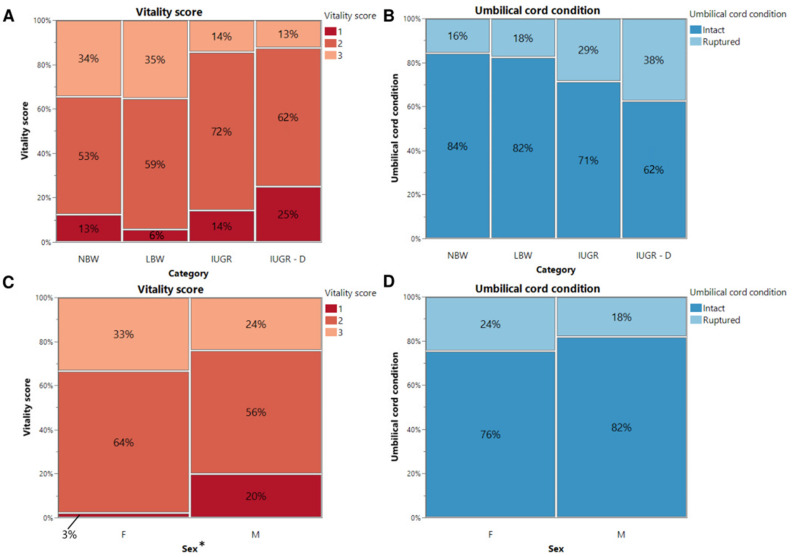
Vitality score (**A**,**C**) and umbilical cord condition (**B**,**D**) at birth. Percentages of normal-birth-weight piglets (NBW), low-birth-weight piglets (LBW), intra-uterine-growth-restricted piglets that were still alive after 24 h (IUGR), and intra-uterine-growth-restricted piglets that were dead within 24 h (IUGR-D) are shown on the mosaic plots. Significant differences between the categories or sex (linear mixed models, Tukey post-hoc analysis, *p* ≤ 0.05) are indicated by an asterisk (*).

To differentiate between IUGR piglets that survived beyond 24 h and those that did not, the piglets were categorized as either IUGR alive or IUGR dead piglets. There was no significant interaction between sex and birth weight category regarding vitality score (Figure 1A,C) and umbilical cord appearance (Figure 1B,D) (*p* = 0.571 and *p* = 0.529, respectively).

Referring to Figure 1A, of 32 NBW piglets, 4 received a score of 1 (13%), 17 received a score of 2 (53%), and 11 received a score of 3 (34%). Of the 34 LBW piglets, 2 had a score of 1 (6%), 20 had a score of 2 (59%), and 12 had a score of 3 (35%). Of the 21 IUGR piglets that were still alive after 24 h, 3 had a score of 1 (14%), 15 a score of 2 (72%), and 3 a score of 3 (14%). Finally, of the 8 IUGR piglets that died within 24 h, 2 were rated with a score of 1 (25%), 5 with a score of 2 (62%), and 1 with a score of 3 (13%). None of the LBW, IUGR alive, or IUGR dead piglets scored 4. Regarding the umbilical cord appearance (Figure 1B), it was intact for 27 NBW (84%), 28 LBW (82%), 15 IUGR alive (71%), and 5 IUGR dead (62%) piglets after birth. However, it was ruptured in 5 NBW (16%), 6 LBW (18%), 6 IUGR alive (29%) and 3 IUGR dead (38%) piglets. Among NBW, LBW, and IUGR piglets (IUGR alive and IUGR dead piglets), neither vitality score nor umbilical cord appearance showed significant differences (*p* = 0.085 and *p* = 0.280, respectively).

Looking at Figure 1C, 15 (33%) of the female piglets received a score of 1, 29 (64%) of the female piglets received a score of 2, and only 1 (3%) of the female piglets received a score of 3. For the male piglets, 12 (24%) received a score of 1, 28 (56%) received a score of 2, and 10 (20%) received a score of 3. For the umbilical cord appearance, it was noted that 11 (24%) of the female piglets had a ruptured umbilical cord, and 34 (76%) had an intact umbilical cord. For the male piglets, 9 (18%) of the umbilical cords were ruptured, and 41 (82%) were still intact after birth. However, no significant difference between males and females was noted in umbilical cord appearance (*p* = 0.401). Interestingly, a substantial difference in vitality score was observed between sexes (*p* = 0.044). More females had a vitality score of 2 or 3, while fewer females had a vitality score of 1, when compared with the male piglets.

### 3.3. Body Temperature

**Figure 2:**
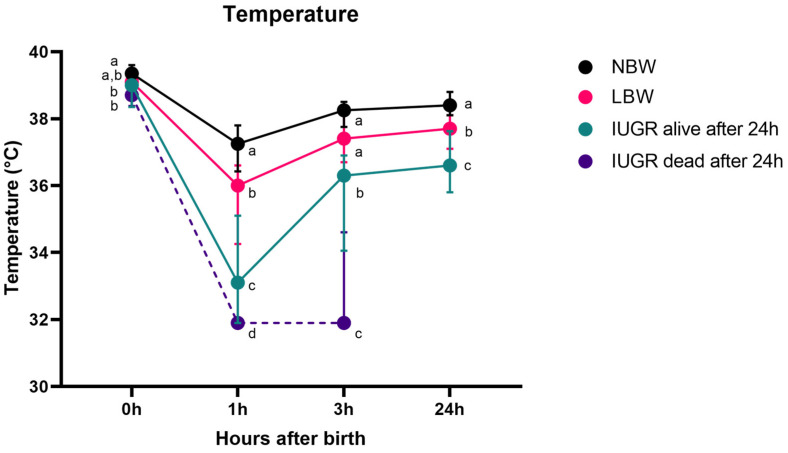
Rectal temperature over 24 h (median (IQR)). Normal-birth-weight piglets (NBW; black line, *n* = 32), low-birth-weight piglets (LBW; pink line, *n* = 34), intra-uterine-growth-restricted piglets that were still alive after 24 h (IUGR alive; green line, *n* = 21) and intra-uterine-growth-restricted piglets that were dead within 24 h (IUGR dead; purple line, *n* = 8) are shown on the graph. Significant differences between the categories per time point (linear mixed models, Tukey post-hoc analysis, *p* ≤ 0.05) are indicated by a different letter (a–d). The median (point) and the IQR (error bars) are shown for each group.

Figure 2 shows the evolution of body temperature over the first 24 h for NBW, LBW, IUGR alive, and IUGR dead piglets. There was no interaction between sex and category (*p* = 0.590) and between sex and timepoint (*p* = 0.471). Interestingly, the interaction term between category and timepoint was significant (*p* < 0.0001). No significant difference was observed between male and female piglets (*p* = 0.196).

The general trend of the rectal temperature in piglets is as follows: they are born with a high temperature. One hour after birth, the temperature notably decreases in NBW, LBW, and IUGR piglets that survive beyond 24 h and IUGR piglets that do not survive (*p* < 0.0001 for all categories). Following this decline, the rectal temperature rises gradually, resulting in a significantly higher temperature three hours post-partum for NBW, LBW, and IUGR piglets that survive beyond 24 h (*p* < 0.0001 for all categories). However, unlike their counterparts, the rectal temperature of IUGR piglets that do not survive do not show this increase (*p* = 0.640). The body temperature of all piglets at 24 h remains lower than at birth (*p* < 0.0001 for all categories).

At birth, NBW piglets and LBW piglets had the highest rectal temperatures of 39.4 (38.9–39.6) °C and 39.1 (38.6–39.5) °C, respectively. The rectal temperatures at birth between NBW and LBW piglets were not significantly different (*p* = 0.150). IUGR alive piglets registered a rectal temperature of 39.0 (38.4–39.2) °C, markedly lower than NBW (*p* = 0.004) but not different from LBW (*p* = 0.307) piglets. IUGR dead piglets had the lowest rectal temperature, 38.7 (38.4–38.9) °C, distinctly cooler than NBW, but not LBW and IUGR alive piglets (*p* = 0.014, *p* = 0.236, *p* = 0.916, respectively).

One hour after birth, NBW piglets exhibit a temperature of 37.3 (36.4–37.8) °C, being higher than the rectal temperature of LBW piglets (36.0 (34.3–36.6) °C, *p* = 0.009) and IUGR alive piglets (33.1 (31.9–35.1) °C (comparison with NBW: *p* < 0.0001, comparison with LBW: *p <* 0.0001). IUGR dead had the lowest rectal temperature (31.9 (31.9–31.9) °C (comparison with IUGR: *p* = 0.010, comparison with NBW and LBW: *p* < 0.0001).

Three hours post-partum, NBW piglets had the highest temperature at 38.3 (37.8–38.5) °C, which was not significantly higher than the rectal temperature of LBW piglets at 37.4 (36.7–38.1) °C (*p* = 0.230). However, it was higher than the body temperature of IUGR alive piglets with a rectal temperature of 36.3 (34.1–36.9) °C (comparison with NBW and LBW: *p* < 0.0001). IUGR dead had the lowest rectal temperature (including IUGR piglets that were alive after one day) (31.9 (31.9–34.9) °C (comparison with NBW, LBW and IUGR alive, *p* < 0.0001).

24 h post-partum, NBW piglets had a rectal temperature of 38.4 (38.1–38.8) °C, LBW piglets 37.7 (37.1–38.1) °C, and IUGR piglets 36.6 (35.8–37.6) °C. The difference between NBW and LBW was significant (*p* = 0.018), as it was between NBW and IUGR (*p* < 0.0001) and between LBW and IUGR (*p* = 0.001).

### 3.4. Latency

There was no interaction between sex and birth weight category regarding the latency data (interaction term latency to reach udder: *p* = 0.481; interaction term latency to reach a functional teat: *p* = 0.800; interaction term latency to suckle: *p* = 0.657). No notable differences were found related to sex for latency to reach udder (*p* = 0.628), latency to reach a functional teat (*p* = 0.396), and latency to suckle (*p* = 0.404).

**Figure 3:**
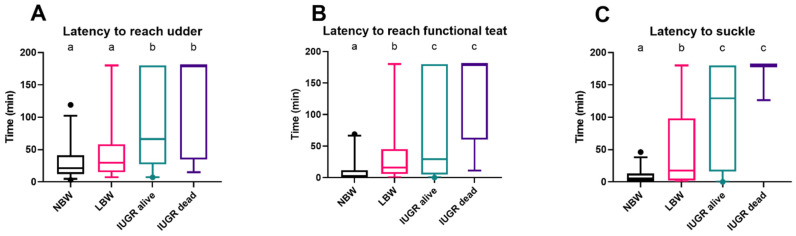
Latency to reach the udder (**A**), a functional teat (**B**), and to suckle (**C**), with a 3 h cutoff. Normal-birth-weight piglets (NBW; black box, *n* = 32), low-birth-weight piglets (LBW; pink box, *n* = 34), intra-uterine-growth-restricted piglets that were still alive after 24 h (IUGR alive; green box, *n* = 21) and intra-uterine-growth-restricted piglets that were dead within 24 h (IUGR dead; purple box, *n* = 8) are shown in the boxplots. Significant differences between the categories (linear mixed models, Tukey post-hoc analysis, *p* ≤ 0.05) are indicated by a different letter (a–c). For each group, the median (tick line), the IQR (edges boxes), and the 5th and 95th percentiles (whiskers) are shown. The outlier in graphs (**A**–**C**) within the NBW group represents an NBW piglet that took longer to reach the udder, find a functional teat, and begin suckling after birth compared to the other piglets in the group.

NBW and LBW piglets arrive at the udder more quickly than IUGR piglets (*p* = 0.0003 and *p* = 0.022). There was no difference in reaching the udder regarding LBW and NBW piglets (*p* = 0.079) and between IUGR dead and alive piglets (*p* = 0.092) (Figure 3A). In terms of reaching and touching a functional teat with their snout, NBW piglets were significantly quicker than LBW (*p* = 0.0003) and IUGR piglets (*p* < 0.0001). Only two of the IUGR dead piglets (25%) made it to a functional teat within three hours, compared with most IUGR alive piglets (67%) (*p* = 0.044) (Figure 3B). NBW piglets started to suckle shortly after reaching the functional teat, whereas LBW piglets took longer (*p* = 0.0004). IUGR piglets that were alive started to suckle a lot later than NBW (*p* < 0.0001) and LBW (*p* = 0.017) piglets. Nevertheless, only one IUGR dead piglet suckled; the other IUGR piglets that died within 24 h never suckled during the first three hours after birth (Figure 3C).

Furthermore, it was explored whether early-born piglets had a higher chance of accessing the udder, finding a functional teat, and starting suckling than mid- or late-born piglets (Figure S1). There was no interaction between birth weight category and birth order (interaction term: *p* = 0.463 for udder, *p* = 0.591 for teat, *p* = 0.198 for suckle), as well as between sex and birth order (interaction term: *p* = 0.181 for udder, *p* = 0.718 for teat). Since the interaction term between birth weight category and birth order was not significant, it can be assumed that there was no notable variation in the time taken to reach the udder, find a functional teat, and begin suckling among early-born, mid-born, and late-born piglets across NBW, LBW and IUGR categories. Additionally, sex did not interact significantly with birth order in affecting the time to reach the udder and find a functional teat. However, a significant interaction was observed between sex and birth order concerning the latency to suckle (*p* = 0.033). When examining birth order, it proved insignificant in male piglets (*p =* 0.705). In contrast, a significant variation was found within female piglets (*p =* 0.008). Early born female piglets started to suckle significantly slower compared with mid-born female piglets (*p* = 0.003) but not compared with late-born female piglets (*p* = 0.829). Mid-born female piglets initiated suckling significantly faster than late-born female piglets (*p* = 0.006).

### 3.5. Weight Gain and Colostrum Intake

There was no interaction between sex and category for weight gain (interaction term: *p* = 0.420) and colostrum intake (interaction term: *p* = 0.646).

**Figure 4:**
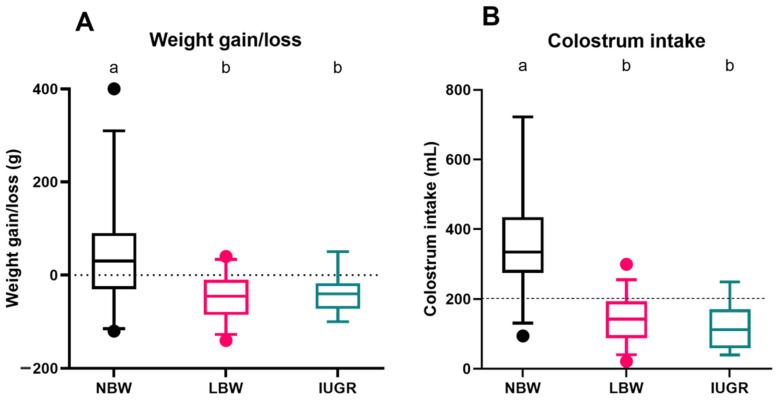
Weight gain/loss (**A**) and colostrum intake (**B**) over a 24 h period. Normal-birth-weight piglets (NBW; black box, *n* = 32), low-birth-weight piglets (LBW; pink box, *n* = 34), and intra-uterine-growth-restricted piglets (IUGR alive; green box, *n* = 21) are shown in the boxplots. For each group, the median (tick line), the IQR (edges boxes), and the 5th and 95th percentiles (whiskers) are shown. The outlier in graph A among the NBW piglets represents a single piglet that showed a higher weight gain compared to the rest of the group. Significant differences between the categories (linear mixed models, Tukey post-hoc analysis, *p* ≤ 0.05) are indicated by a different letter (a–b).

For the surviving NBW (*n* = 32), LBW (*n* = 34), and IUGR (*n* = 21) piglets, weight gain or loss (Figure 4A) and colostrum intake (Figure 4B) were estimated over 24 h. The median weight gain of NBW piglets was 30.0 (−30.0–90.0) g, which was significantly higher than that of LBW (*p* < 0.0001) and IUGR piglets (*p* = 0.0001). Weight loss was observed for both LBW and IUGR piglets, with median weight losses of −45.0 (−85.0–10.0) g and −40.0 (−72.5–20.0) g, respectively, but there was no significant difference between LBW and IUGR piglets (*p* = 0.997). Female and male piglets did not differ in weight gain (*p* = 0.074).

NBW piglets consumed considerably more colostrum than LBW (*p* < 0.0001) and IUGR piglets (*p* < 0.0001), with a median of 334.6 (274.6–434.6) mL. Both LBW (142.2 (88.1–93.5) mL) and IUGR (100.8 (58.9–143.7) mL) had colostrum intakes of less than 200 mL. There was a significant effect of sex on the colostrum intake (*p* = 0.025), where male piglets (142.2 (88.1–260.6) mL) consumed less colostrum than female piglets (200.8 (125.5–344.0) mL).

### 3.6. Principal Component Analysis

Principal component analysis (PCA) was used to explore relationships between the recorded variables. Principal component (PC) 1 accounts for 43% of the total variance and can be primarily attributed to 24 h weight (0.90), birth weight (0.89), rectal temperature 1 h post-partum (0.85), CRL (0.84), and colostrum intake (0.81). Additionally, though slightly lesser, contributions to PC 1 include rectal temperatures 3 h (0.77) and 24 h (0.69) post-partum, as well as weight gain (0.57). Negative influences on PC 1 are seen with the category code (−0.87) and, to a lesser extent, latency measurements such as time to reach the udder (−0.66), time to find a functional teat (−0.70), and time to start suckling (−0.75). PC 2, explaining 13% of the variation, is notably affected by PI (0.87) and BMI (0.81). PCs 1 and 2 collectively explain 56% of the overall variance in the data.

**Figure 5:**
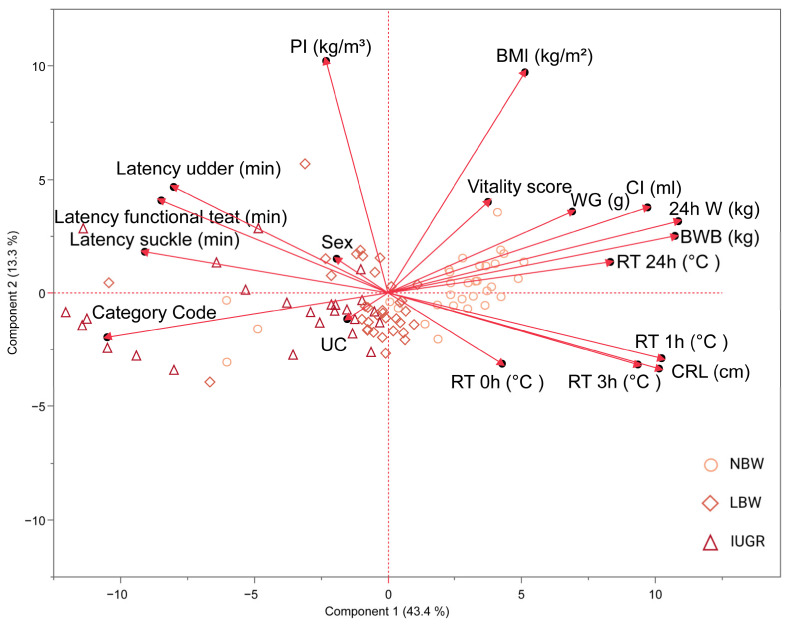
Visualization of the first two principal components (PC) in a biplot from principal component analysis (PCA) 1. PCA has been carried out on normal-birth-weight piglets (NBW; circles, *n* = 32), low-birth-weight piglets (LBW; rhombuses, *n* = 34), and intra-uterine-growth-restricted piglets (IUGR alive; triangles, *n* = 21) and is shown on this biplot. The PCA included 24 h weight (24 h W); body weight at birth (BWB); weight gain (WG); colostrum intake (CI); body mass index (BMI); ponderal index (PI); crown–rump length (CRL); rectal temperature at birth (RT 0 h), 1 h (RT 1 h), 3 h (RT 3 h) and 24 h (RT 24 h) post-partum; latency to reach the udder (latency udder), a functional teat (latency functional teat), and to begin suckling (latency suckle); umbilical cord appearance (UC); vitality score; sex; and category (category code).

Figure 5, depicted through PC 1 and PC 2 in the PCA, highlights a strong correlation among several variables: weight gain, colostrum intake, birth weight, 24 h weight, and rectal temperature at 24 h post-partum. The observed negative correlation with the category code (where IUGR = 3, LBW = 2, and NBW = 1) is consistent with our selection procedure, indicating that higher category codes are associated with lower weights. As expected, a significant correlation exists among latency data. While PI and BMI correlate, as they are both variables that represent ‘size,’ this is not as pronounced. Moreover, rectal temperatures at 1 h and 3 h post-partum strongly correlate with CRL. Logically, these variables show a negative correlation with latency data, suggesting that the longer it takes piglets to reach the udder and other early post-birth milestones, the lower their rectal temperatures and CRL measurements. Vitality score, sex, umbilical cord appearance, and rectal temperature at birth have a minimal impact on PCA 1.

The cluster of IUGR piglets is primarily centered around latency measures, category code, and the appearance of the umbilical cord. Conversely, IUGR piglets are far from vectors like weight metrics, BMI, and rectal temperature. On the other hand, NBW piglets cluster around health and growth metrics, such as weight at birth, 24 h weight, weight gain, colostrum intake, BMI, and rectal temperature at 24 h post-partum, and are positioned distantly from latency measures. LBW piglets tend to be centered in the middle.

## 4. Discussion

In the early stages of life, particularly the first 24 h post-partum, neonatal piglets face a period of high vulnerability, marked by significantly elevated mortality rates [40]. In addition, the groundwork is laid for the piglet’s future growth, health, and overall wellbeing during this period and even before birth. Accordingly, insights into the strengths and vulnerabilities of piglets during these early stages can significantly enhance intervention strategies. This observational study provides a comprehensive overview of various neonatal piglet parameters, including body measurements, physiological responses, and early behaviors, such as moving to the udder and suckling colostrum within the first 24 h post-birth, displayed by NBW, LBW, and IUGR piglets.

Initially focusing on conditions at birth, we found that, while IUGR piglets were likelier to have lower vitality scores, there were no significant vitality differences compared with LBW and NBW piglets. This aligns with findings of Rootwelt et al. [41], who have suggested that low birth weight does not directly correlate with decreased vitality. Despite this, it remains imperative to explore other factors at birth that might affect vitality. One critical factor is the state of the umbilical cord at birth. A ruptured umbilical cord can lead to oxygen deprivation in the affected piglets and lower vitality at birth [35,41]. Our observations highlight the incidence of ruptured umbilical cords among IUGR, NBW, and LBW piglets. Ruptured umbilical cords were observed in all categories and among all vitality scores, indicating that such incidents do not necessarily result in lower vitality scores at birth. However, the location of the umbilical cord rupture could play a role. A rupture near the piglet likely results in more blood loss and reduced oxygen supply, significantly affecting the viability score. Conversely, if the rupture leaves a longer piece of the umbilical cord attached to the piglet, it tends to have less impact on its viability [42]. However, in this study, all piglets had an umbilical cord longer than 5 cm, which explains why no effect on vitality was observed.

Another important aspect at birth is the morphotype of a piglet. In our study, IUGR piglets displayed significantly lower birth weights, shorter CRL, and, consequently, lower BMI compared with LBW and NBW piglets, aligning with findings from previous research [5,6,12,43,44]. However, while these studies have noted a lower PI in IUGR piglets compared with their littermates, we did not observe this [5,6]. Larger piglets with higher weight, BMI, and PI typically outperform their smaller, low-birth-weight counterparts in the competition for access to a functional teat [6,42]. Hales et al. [28] further support this, showing that a piglet with a higher BMI is more likely to survive among two piglets of similar weight. Baxter et al. [15] have demonstrated that birth weight alone is not a definitive predictor of piglets’ viability and survival, highlighting the way in which BMI and PI are more accurate when forecasting prenatal survival. Similarly, Tucker et al. [45] have found that morphological measures, such as BMI and abdominal circumference, could better predict piglet viability, affecting their performance and survival, than merely considering weight. Based on these findings, it is not surprising that mortality rates were elevated in this study in IUGR piglets compared with those in the LBW and NBW categories. However, the higher mortality rates in IUGR piglets cannot be solely attributed to morphological measures [1,40,45].

Another aspect of postnatal survival is the ability of piglets to establish thermal balance. Thermoregulation is a crucial factor in neonatal survival as piglets are delivered wet into an environment much colder than uterine conditions [31]. Furthermore, newborn piglets have little adipose tissue and lack brown fat, leading to a very low energy reserve for thermoregulation [46]. Consequently, they depend on non-shivering thermogenesis from muscles, which quickly depletes their limited energy reserves [31]. Additionally, due to their greater surface-to-body mass ratio, small piglets, including those with IUGR, face a higher heat loss and a diminished capacity for thermoregulation compared with NBW piglets [31,42,46]. Indeed, rectal temperature measurements from the different birth weight categories indicated a decrease one hour after birth. LBW and IUGR piglets showed a reduced ability to recover their temperatures compared with NBW piglets. Notably, IUGR piglets’ rectal temperatures were lower than those of LBW piglets. This aligns with Schmitt et al. [30], who suggested that the level of IUGR might hinder the piglets’ ability to regulate their body temperature within the first hour after birth. Consequently, the severe IUGR piglets in this study had the lowest temperatures, whereas LBW piglets, showing mild or no signs of intra-uterine growth retardation, had higher temperature values in the first hours following birth [30]. This can be explained by our observation that IUGR and LBW piglets require more time to ingest enough colostrum. Colostrum intake is pivotal in providing a piglet the necessary energy to achieve thermal stability. It has been observed that a rise in blood glucose levels is only apparent 4 h after birth, which explains why we were unable to observe a significant rise in body temperature after 3 h post-partum [42,47]. Additionally, IUGR piglets that died within the first 24 h exhibited rectal temperatures at 1 h post-partum below the minimal critical temperature of 33–35 °C, the temperature below which piglets need to increase heat production to maintain a homeothermic balance [42]. According to Herpin et al. [46], body temperatures below this critical threshold no longer increase shivering to generate heat. Because newborn piglets have limited glycogen reserves, which can meet their energy needs only for 7 to 8 h [42], a lack of colostrum intake leaves them vulnerable to severe hypothermia. Failure to obtain adequate colostrum may lead to starvation and hypothermia, increasing the risk of being overlaid by the sow, known as the chilling–starvation–crushing cycle [42].

To ensure postnatal survival, piglets must perform crucial landmark behaviors, such as moving to the udder and suckling colostrum. Several studies have proposed that piglets must ingest at least 200 mL of colostrum [5,48,49,50]. Colostrum provides more than just energy; it also plays a critical role in piglet immunological defense by supplying immunoglobulins [51,52,53]. Additionally, colostrum supports the maturation of the gut, which is crucial in the first few hours after birth [29,50]. Our findings indicate that NBW piglets move to the udder, find a functional teat, and start suckling quickly after birth. This behavior leads to significant colostrum consumption and is associated with noticeable weight gain within 24 h. LBW piglets, especially IUGR, had difficulties gaining weight after 24 h and had a colostrum intake lower than 200 mL. The reason for this is that they had problems starting suckling. LBW piglets are as quick as a NBW piglet to reach the udder, but finding a teat took longer for them. Consequently, the start of suckle was significantly delayed in LBW piglets. This indicates that LBW piglets fail to compete with NBW for teat access. Another explanation could be that LBW piglets lack the necessary muscular force, impacting their ability to find a teat and start suckling effectively [54]. This issue is compounded by the fact that LBW piglets start life with limited energy reserves, leading to adaptations in their locomotion aimed at greater energy efficiency. As a result, they exhibit lower motor performance when compared with NBW piglets [55]. In this study, IUGR piglets experienced an even more challenging start, showing delayed movement towards the udder and struggling to locate and latch onto a functional teat. These difficulties were compounded by competition with NBW and LBW piglets, often resulting in them being pushed away before they could start suckling. Beyond the size disadvantages, this delay may also be attributed to a delay in neurodevelopment, such as reduced myelination and dendritic development [56]. Further research is required to better understand the mechanism of neural immaturity in IUGR piglets. Mielke et al. [57], studied the locomotion pattern in LBW piglets and found that LBW piglets experience a 4 h delay in the maturation of locomotion patterns. This delay in locomotion development is likely not due to neuronal deficits but more likely related to depleted energy reserves [57,58], as also demonstrated by Vanden Hole et al. [47]. However, their studies made no distinction between LBW and IUGR piglets. This energy deficit, more pronounced in IUGR piglets than in their healthier counterparts, might result not only from smaller initial energy stores [59] but also from a faster depletion rate because of its use in creating a thermostable environment and to move around. Consequently, these piglets may lack the vigor to compete with littermates and to engage in normal feeding behaviors. The question of whether the birth order influenced the latency data was also investigated. However, our study found no significant differences in udder access, functional teat finding, or suckling initiation among early, mid, and late-born piglets across all categories.

PCA was used to explore the relationship between variables. This revealed the distinct clustering of IUGR and NBW piglets on opposite sides. Specifically, NBW piglets were found to be associated with vectors linked to weight metrics and temperature data, which is expected as weight parameters were part of the selection criteria. In contrast, IUGR piglets were found to be closer to vectors related to feeding behavior and stress indicators, such as the appearance of the umbilical cord at birth, and were located away from the temperature and weight metric vectors. This clustering suggests potential risk factors for IUGR piglets, such as delayed suckling or temperature regulation difficulties, which require targeted interventions. In contrast, NBW piglets, clustering around growth and temperature metrics, display robust health and development signs. They also show a reduced association with latency measures, indicating a lower risk of developmental delays or complications that are more common in IUGR or LBW piglets. LBW piglets tend to position between the IUGR and NBW clusters, sharing characteristics with both groups. They may not display the extreme values seen in either group, suggesting they face growth challenges like IUGR piglets while showing healthier physiological metrics like those seen in NBW piglets.

The data presented in this study underscore the necessity for targeted management strategies to support neonatal piglets, particularly those classified as LBW or IUGR. Effective intervention strategies include ensuring adequate heat provision, facilitating sufficient colostrum intake, and implementing fostering practices when necessary. Heat provision is ensured via additional heat sources, such as heat lamps or heated creep areas, or by increasing assistance to newborn piglets, such as by drying and/or moving them to a heated area or the udder soon after birth [43,44]. Along with additional heat sources, intraperitoneal injection with warm saline has shown promising outcomes, as proven by Tucker et al. [60], in enhancing the rectal temperature and survival rates of piglets during their first 24 h of life and up to weaning. Facilitating sufficient colostrum intake can be achieved using strategies such as split suckling (where larger piglets are temporarily removed to allow smaller piglets better access to the teat), cross-fostering (where certain piglets are moved to another sow to ensure equalized litters and piglet sizes), nurse sows (sows that specifically adopt and nurse another litter after weaning their own) and artificial milk provision (where piglets are taken away from their dam and housed until weaning in a special facility with heat lamps and feeding with a milk replacer) [12,40,43,49]. However, strategies aimed explicitly at IUGR piglets may decrease mortality rates in litters from hyper-prolific sows. One strategy to reduce the problem of IUGR piglets involves genetic selection against traits associated with this condition, such as low birth weight or specific piglet morphologies like the “dolphin-shaped” head. However, despite efforts to increase overall birth weight and reduce birth weight variability, the amount of IUGR piglets has not decreased, suggesting the need to explore other related phenotypic traits for selection [12]. Matheson et al. [10] have found that selecting against IUGR can be effective at the maternal level by focusing on the proportion of IUGR-affected piglets within a litter. However, direct selection against the IUGR head morphology in piglets tends to yield minimal and slow progress [10]. Implementing targeted strategies for IUGR piglets, combined with individual colostrum and energy dosing, has shown promising outcomes. For instance, administering glucose injections at birth to serve as an immediate energy source, providing warmth for one hour and placing piglets with a nurse sow to minimize competition significantly boost the growth of IUGR piglets [59]. Despite the promising results of subcutaneous glucose injections, this intervention strategy requires trained personnel or veterinarians and carries risks associated with injection [59,61]. Another targeted strategy for energy supplementation in IUGR piglets is drenching, which involves the oral administration of various substances to individual piglets [25,33,61]. This method has the advantage of providing extra colostrum or milk to low viability IUGR and LBW piglets, giving them a boost for a very brief period during the first day of life [25]. However, despite its potential, results have often been inconsistent and have not consistently improved the long-term survival or performance of IUGR and LBW piglets [25,34]. In addition, the effectiveness of these strategies varies with the piglet’s IUGR status; therefore, humanely killing severely low-viable IUGR piglets is also a critical management practice, preventing unnecessary suffering and ultimately improving animal welfare [12,62].

Despite the effectiveness of current strategies, it is important to carefully consider the feasibility of these strategies, concerning labor demands and cost implications. While interventions such as additional heat sources and glucose injections, as well as drenching, are highly effective, they necessitate increased labor input and trained personnel, which are often in short supply and costly [43,61,63]. To address this, farms may consider automation where feasible, such as using automated heat lamps or creep areas that maintain consistent temperatures with minimal human intervention [64]. Moreover, cross-fostering and nurse sows, while labor-intensive, could be streamlined by careful planning and selective implementation, ensuring that these practices are used only when necessary to reduce labor requirements [43,63]. In addition, it is crucial to recognize the practical challenges associated with these interventions. The use of nurse sows, for example, necessitates the relocation of newborn piglets to another sow after obtaining colostrum from their mother. This strategy, while beneficial, can prolong the lactation period for nurse sows and potentially disrupt the ‘all-in-all-out’ system, increasing the risk of pathogen transfer between batches [43,61,63]. Artificial rearing, while providing a controlled environment for piglets, requires significant financial investment in infrastructure and may not comply with current legislative frameworks [61,63]. Split suckling, on the other hand, has been found to improve colostrum access for smaller piglets by temporarily separating them from larger, more vigorous littermates. While effective, this method also requires careful management and labor to ensure all piglets are properly rotated and fed [43,63]. Similarly, milk cups can be installed to provide supplementary feeding. Nevertheless, the effectiveness of these systems depends on piglets learning to use them (if a system is used that requires activation by the piglet to fill the milk cup) [43,61]. However, another option is a costly automated system that distributes milk from a central point throughout the entire farrowing room. At the same time, a more affordable but labor intensive alternative requires manual refilling of the cups [61,65]. In addition, providing supplementary milk through milk cups may not fully address the competitive dynamics within litters, as larger piglets often dominate access to both the sow’s milk and the supplementary feed, leaving LBW and IUGR piglets at a disadvantage [65,66]. Another approach is the integration of low-cost, low-labor interventions that can be easily implemented, such as optimizing the design of farrowing crates to improve thermal environments naturally or adjusting feeding protocols to ensure that sows produce more nutrient-rich colostrum [43,64]. Additionally, enhancing genetic selection strategies to focus not only on IUGR traits but also on improving overall sow health and litter outcomes could reduce the prevalence of IUGR piglets over time, thus lessening the need for intensive interventions [43]. Further research into new approaches that can reduce costs and labor while specifically targeting IUGR piglets is essential for enhancing outcomes. Understanding and addressing the complexities of IUGR will lead to improved survival rates and the overall welfare of piglets.

## 5. Conclusions

In conclusion, our in-depth study has vividly illustrated the distinct challenges IUGR piglets face compared with their LBW and NBW counterparts. The significantly lower birth weights, shorter CRL, and reduced BMI of IUGR piglets not only delineate their immediate physical disadvantages but also underscore a marked vulnerability in their early life stages, as is shown by lower rectal temperatures and experienced delays in commencing early feeding. The evidence revealed in this study underscores the urgent need for specially tailored care strategies for IUGR piglets to dramatically improve their survival and development by addressing challenges in thermoregulation and initial feeding. This tailored approach not only aligns with ethical standards of animal care but also stands to boost economic outcomes by increasing the viability of the most vulnerable piglets. Thus, this study deepens our understanding of neonatal vitality and sets a clear directive for future management and research focused on optimizing care for at-risk neonatal populations.

## Figures and Tables

**Table 1:** Vitality score evaluated from the first 15 s until 1 min after expulsion ^1^.

Vitality Score	Description
0	Piglet shows no movement and no breathing after 15 s (stillborn).
1	Piglet shows no movement after 15 s, is breathing or attempts are made to breath (coughing, spluttering, clearing its lungs).
2	Piglet shows some movement and attempts are made to right themselves onto the sternum within 15 s, is breathing or attempting to breath.
3	Piglet shows good movement and good breathing. Piglet rights itself onto sternum and attempts are made to stand within 15 s.
4	Piglet shows good movement and good breathing. Piglet stands within 1 min.

^1^ Adapted from Baxter et al. [15,17].

**Table 2:** Piglet characteristics for normal-birth-weight piglets (NBW), low-birth-weight piglets (LBW), and intra-uterine-growth-restricted piglets (IUGR) ^1^.

	NBW	LBW	IUGR	*p*-Value
*n*	32 (16 ♂–16 ♀)	34 (18 ♂–16 ♀)	29 (16 ♂–13 ♀)	
BWB, kg	1.30 ^a^ (1.12–1.44)	0.75 ^b^ (0.70–0.78)	0.57 ^c^ (0.49–0.63)	<0.0001
CRL, cm	26.0 ^a^ (25.0–28.0)	22.0 ^b^ (21.0–23.5)	20.0 ^c^ (18.0–21.8)	<0.0001
BMI, kg/m^2^	18.4 ^a^ (17.0–21.1)	15.3 ^b^ (14.0–17.5)	14.3 ^c^ (12.7–15.3)	<0.0001
PI, kg/m^3^	68.0 ^a^ (63.1–80.5)	69.9 ^a^ (58.0–85.2)	71.3 ^a^ (56.3–83.2)	0.805
Mortality, %	0 ^a^	0 ^a^	31 ^b^	<0.0001

^1^ The table presents the number of piglets per birth weight category and sex (*n*), along with the median and interquartile range (IQR) of body weight at birth (BWB), crown–rump length (CRL), body mass index (BMI), ponderal index (PI) (linear mixed models). *p*-values are reported with significance set at *p* ≤ 0.05. Tukey post-hoc analysis was performed, and a different letter (a–c) indicates significant differences between the categories. BMI was calculated as body weight/CRL^2^. PI was calculated as body weight/CRL^3^.

## Data Availability

All data presented in this study are contained within the article.

## Supplementary Materials

The following supporting information can be downloaded at https://www.mdpi.com/article/10.3390/ani14172508/s1, Figure S1: Latency to reach the udder (A), a functional teat (B) and to suckle (C), for the different birth order categories.
