# Supplementary material for: Thriving or Striving: Comparing Intra-Uterine Growth Restricted, Low Birth Weight and Normal Birth Weight Piglets within the First 24 Hours
Source: Animals (Basel). 2024 Aug 29;14(17):2508. doi: 10.3390/ani14172508 (PMC11394454; doi:10.3390/ani14172508)
Supplement: Supplementary file 1 [file animals-14-02508-s001.zip › animals-3122651-supplementary.pdf]

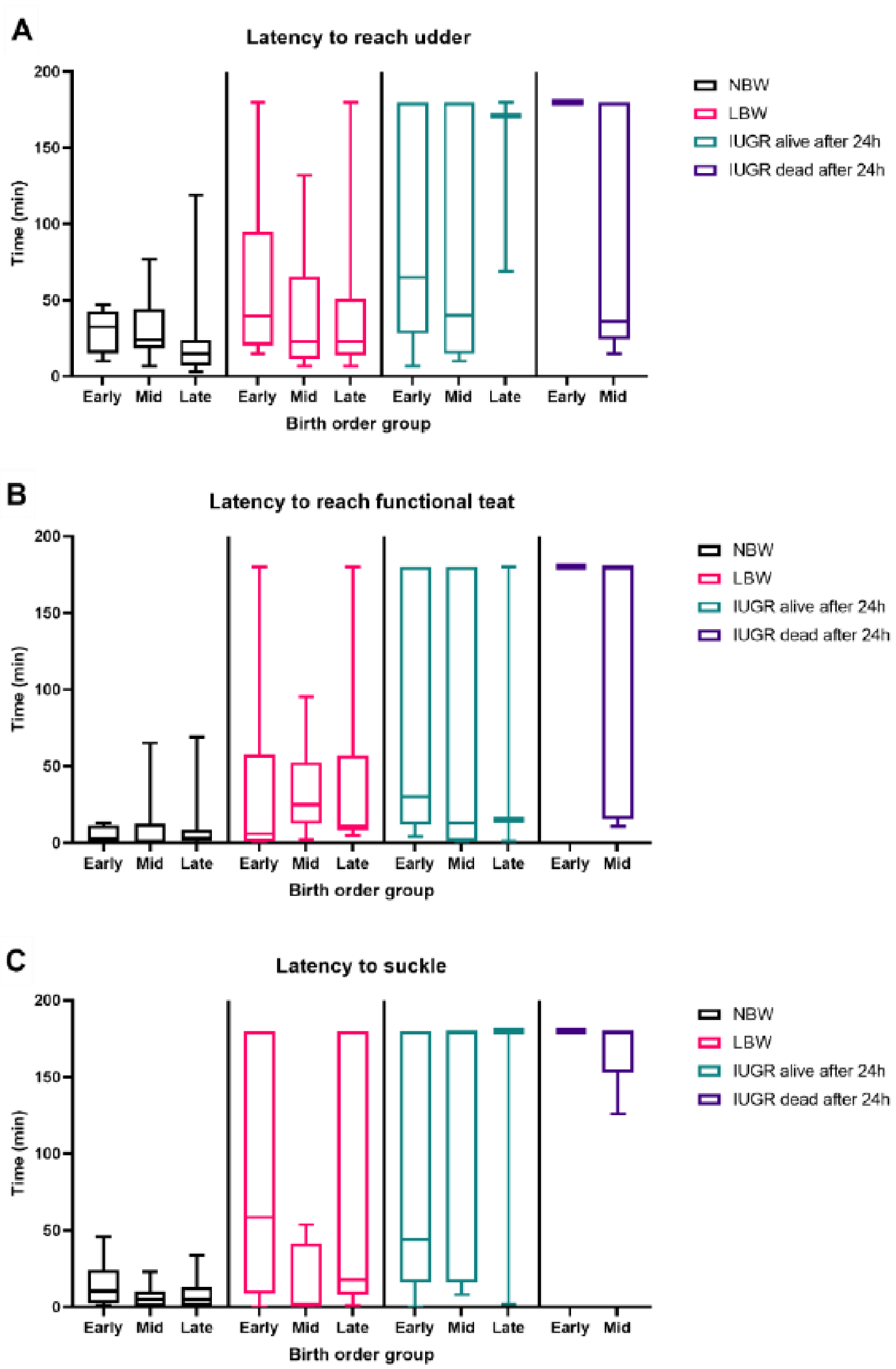

Figure S1. Latency to reach the udder (A), a functional teat (B) and to suckle (C), for the different birth order categories. Normal birth weight piglets (NBW; black boxes, N = 32), low birth weight piglets (LBW; pink boxes, N = 34), intra-uterine growth restricted piglets that were still alive after 24 hours (IUGR alive; green boxes, N = 21) and intra-uterine growth restricted piglets that were dead within 24 hours (IUGR dead; purple boxes, N = 8) are shown in the boxplots. There were no significant differences between the birth order groups within a birth weight category (interaction term: $p = 0.463$ for udder, $p = 0.591$ for teat, $p = 0.198$ for suckle). For each group, the median (tick line), the IQR (edges boxes), and the 5th and 95th percentiles (whiskers) are shown.
